# GPs’ experiences of diagnosing and managing childhood eczema: a qualitative study in primary care

**DOI:** 10.3399/bjgp18X694529

**Published:** 2018-02-16

**Authors:** Emma Le Roux, Kingsley Powell, Jonathan P Banks, Mathew J Ridd

**Affiliations:** Centre for Academic Primary Care, School of Social and Community Medicine, University of Bristol, Bristol.; Centre for Academic Primary Care, School of Social and Community Medicine, University of Bristol, Bristol.; National Institute for Health Research Collaborations for Leadership in Applied Health Research and Care West (NIHR CLAHRC West), University Hospitals Bristol NHS Foundation Trust, Bristol, UK.; Centre for Academic Primary Care, School of Social and Community Medicine, University of Bristol, Bristol.

**Keywords:** atopic eczema/dermatitis, eczema, paediatrics, primary health care, qualitative research

## Abstract

**Background:**

Eczema is common among children, and in the UK the majority are managed by GPs. The most common cause of poor disease control is incorrect use of topical treatments. There is a lack of research into the challenges faced by GPs in diagnosing and managing this condition.

**Aim:**

To explore the experiences of GPs in assessing and managing children with eczema.

**Design and setting:**

Qualitative study in primary care in England.

**Method:**

Semi-structured interviews with 15 GPs were audiorecorded, transcribed verbatim, and analysed thematically using the framework method.

**Results:**

GPs described a paucity of dermatology training. Although most GPs were confident diagnosing uncomplicated eczema, they reported using a trial-and-error approach to prescribing emollients, and were uncertain about quantities of topical treatments to issue. Mild and moderate potency topical corticosteroids (TCS) were commonly used, but most GPs lacked confidence in recommending potent TCS, and viewed parents or carers to be fearful of using all strengths of TCS. GPs perceived adherence to treatments to be low, but provision of information to support self-care was variable. Routine review of medication use or disease control was uncommon, which GPs attributed to service constraints. Participants’ views on the causes and management of eczema were perceived to be at odds with parents and carers, who were said to be overly focused on an underlying cause, such as allergy.

**Conclusion:**

GP uncertainty in managing eczema, lack of routine information and review, and perceived dissonance with parents around causation and management may be contributing to low concordance with treatments.

## INTRODUCTION

Eczema is the most common chronic inflammatory skin disease and, after skin infections, the most frequent reason for a new dermatological consultation in general practice.^1^ The majority of children with eczema have mild to moderate disease and are managed in primary care by GPs. It can place a heavy burden on the affected child and their family,^2^^,^^3^ and requires a high level of self-management.^4^

There is a paucity of studies examining the management of patients with eczema in primary care. Evidence suggests that emollients are underused^5^^,^^6^ and, historically, the majority of children referred to dermatology outpatient clinics have mild-to-moderate disease which could be managed in primary care.^7^ The literature suggests that the most common reason for treatment failure is non-adherence to topical treatments.^8^ Reasons for this have been identified in qualitative studies with parents and carers (hereafter parents) and include: under appreciation by healthcare professionals of their child’s condition, confusion about eczema and its treatment regimens, and fears over medication side-effects, especially topical corticosteroids (TCS).^9^^–^12 Patients’ perception of ‘dismissive’ and ‘unsympathetic’ attitudes by GPs have been reported,^13^ and a survey from the National Eczema Society in 1993 found that patient expectations had only been completely met in 12% of GP consultations.^14^ However, the perspective of GPs on the management of childhood eczema is not known.

As part of a wider study developing a written action plan (WAP) to support self-management by parents and children,^15^ the authors sought to explore GPs’ experience and confidence in the diagnosis and management of children with eczema.

## METHOD

### Recruitment

GPs were recruited from practices participating in the authors’ eczema WAP study, professional networks, and by word of mouth.^16^ All responders completed an online screening questionnaire. Purposive sampling^17^ was used to recruit a range of participants by sex, years in role, and sociodemographic area of practice.

### Interviews

GPs gave written informed consent to participate, and a reimbursement of £50 was offered for their time. Semi-structured interviews were carried out in participants’ practices, homes, and by telephone between September 2016 and January 2017. An interview topic guide based on the aims of the study and the available research literature was piloted, employed, and revised during the study as new issues arose (Box 1). Interviews lasted between 30 and 68 minutes, were audiorecorded, transcribed verbatim, checked, and anonymised. Reflexive field notes were made following the interviews.

**How this fits in**

In the UK, GPs diagnose and manage most children with eczema, but treatment failure is common due to low concordance with topical treatments. GPs in this study expressed uncertainties in prescribing emollients and topical corticosteroids, and proactive review of patients was uncommon. Reaching a shared treatment plan with carers may be hampered by differences between carer and professional views of the role of allergy in eczema causation. Clear treatment guidance by GPs, along with proactive review, may help address parents’ concerns and improve outcomes.

Box 1. **Interview topic guide**

- Professional background, current role, years in role, clinical interests and responsibilities, undergraduate and vocational training in dermatology, access to GP with specialist interest in dermatology, practice demographics.
- What resources do you use to support you with diagnosing and managing eczema?
- Can you describe your experience of looking after children with eczema, including recent case review? (Frequency of encounters, medications issued, difficulties encountered around diagnosis, treatment, or advice given).
- Have you managed any cases of infected eczema? (Advice and treatment offered).
- How often are you aware of parents or carers using complementary and alternative therapies?
- What is your approach to parent or carer questions around eczema and allergies?
- What follow-up arrangements do you usually make?
- How do you assess compliance with eczema treatments?
- When would you consider referring a child for specialist advice?
- How have you/do you try to support parents/carers in looking after their child?
- Are there any other tools/resources you think might be helpful to improve the care of children with eczema?

### Analysis

Transcripts were imported to NVivo 10 software to facilitate coding and data organisation. Inductive thematic analysis, using a framework approach, was undertaken concurrently with data collection.^18^ A coding frame based on data from the first four GP transcripts was applied to subsequent transcripts and refined iteratively, applying the constant comparative method derived from grounded theory approaches.^19^ The members of the research team met regularly to resolve coding queries, and a subset of interviews (*n* = 3) were analysed independently, with coding discrepancies discussed to maximise rigour and enable further refining of the coding framework. Codes were compared both within and across interviews, and then grouped into key themes and subthemes. Data collection was discontinued once the main themes were developed with no new information emerging (data saturation).^20^

## RESULTS

Expressions of interest were received from 48 GPs in 20 practices. Of the 32 GPs invited to interview, 15 (47%) took part; 13 interviews were undertaken face-to-face and two by telephone.

The 11 female and four male participants had a range of personal characteristics and worked in a variety of practices located in Bristol, Gloucestershire, Portsmouth, and Nottingham, England (Table 1). Most GPs reported working in practices serving mainly white British patients, although several reported having communities of eastern European patients, particularly Polish. One-third of the GPs interviewed work within inner-city practices or had a branch surgery, where they reported seeing a higher proportion of ethnic minorities.

Three main themes emerged: dermatology training, education, and practice; eczema consultations; and GPs’ perception of parental management of eczema.

### Dermatology training, education, and practice

Both recently qualified GPs and those with more experience felt that eczema was generally accorded a low priority in primary care practice, and this was also reflected in a lack of training at undergraduate and postgraduate level:

‘We did a very brief stint on dermatology and, um, I remember quite a few extreme cases. I’m not sure how much I learnt about the sort of dermatology that we see in general practice as an outpatient.’

(GP 14, female partner, 11 years’ experience)

Exceptions to this were two GPs with a specialist interest (GPSI) who had undertaken a diploma in dermatology, and one GP who had undertaken a dermatology position as part of a medical rotation before GP training.

GPs maintained their dermatology knowledge through study days or peer group learning. Some relied on dermatology outpatient letters about their patients as a learning resource:

*‘I tend to learn also what’s the appropriate strength* [of TCS] *by reading the dermatologist’s letter that comes back.’*

(GP 5, female partner, 12 years’ experience)

A minority of GPs said their practice had a GP dermatology lead to whom they would make internal referrals when uncertain about patient management. Rather than using guidelines, GPs turned to professional online resources (for example, www.gpnotebook.co.uk), links from within the clinical software systems, or Google searches for guidance. Websites with photographs were utilised to achieve a shared understanding with the parents of the working diagnosis:

‘Dermnet, it’s my favourite, and I quite like getting the pictures up and saying to patients “right, what do you think, you know, look at this, this looks like yours“, and patient.co.uk as well.’

(GP 8, female salaried, 14 years’ experience)

**Table 1.** Characteristics of participants and their practices (*n* = 15)

**Personal**	
Mean age, years (range)	44 (30–56)
Mean years in role (range)	14 (0.5–29)
GP partner	3 Male, 7 Female
Salaried GP	1 Male, 4 Female
Specialist interest in dermatology	1 Male, 1 Female
Personal experience of eczema (family member affected)	2 Male, 0 Female
Mean self-reported confidence with eczema management, 0 low, 10 high (range)	7.3 (5–9)
**Practice**	
Mean list size (range)	10 441 (5478–18 221)
Mean practice IMD 2015,^a^ 1 low, 10 high (range)	6.2 (1–10)

a Index of multiple deprivation (IMD) 2015 (an overall measure of deprivation experienced by people living in an area).

Some GPs viewed consultations for eczema as an opportunity to catch up if running late. These opinions were coupled with an overall feeling that, due to competing demands of chronic disease management and multimorbidity in an ageing population, dermatology is a low priority in general practice. The impact of childhood eczema and its effect on quality of life were infrequently described:

*‘Dermatology is not really that important in primary care at the moment, I have to say* [laughs]*. There’s much more — there’s the care of the elderly and their management which is preoccupying all of us, getting people out of hospitals or not to get into hospitals.’*

(GP 2, male partner, GPSI, 16 years’ experience)

However, for those GPs with a specialist interest in dermatology, and those with personal experience of eczema, eczema consultations were described as time consuming:

‘The consultation tends to overrun with me because I feel like there’s a lot, you know, when you’re dealing with someone whose eczema’s not well-managed, there’s a lot to cover in 10 minutes.’

(GP 4, male salaried, child with eczema, <1 years’ experience)

### Eczema consultations

#### Diagnosis

Diagnosing eczema in children was not reported to present a challenge to most GPs. Where uncertainty was described, it was in the context of a rash in an atypical distribution or where the differential diagnosis included a fungal or viral infection. There was also varying confidence in recognising bacterial and viral infection complicating eczema, and eczema in different ethnic skin types:

*‘Sometimes, uh,* [pause] *it can be hard to know if they’ve got an infection or not, and sometimes you see them a couple of times and there’s nothing obvious. There’s* [sic] *no pustules or anything like that, and you give antibiotics a go ‘cos it’s a systemic, florid flare, and actually it all settles down.’*

(GP 1, female partner, 13 years’ experience)

‘Black African patients, yeah that can be difficult actually. I’ve not seen it a lot. I don’t know if it’s less common but, um, but yes, you know they’ll show you eczema and you’re thinking gosh, is it?’

(GP 13, female salaried, 17 years’ experience)

### Treatment

Almost half of GPs interviewed said they routinely asked about current skin care regimens, and most expressed an awareness that parents have usually tried over-the-counter topical products, although use of complementary and alternative therapies was said to be uncommon. Though many GPs were aware of parents that disliked the consistency of some emollient treatments, few reported discussing options with parents, or sought agreement about what was acceptable to them before prescribing. With a large range of emollients available, a trial-and-error approach to prescribing emollients was commonplace, with GPs also being uncertain about quantities to issue:

*‘Regarding emollients, I guess the challenging thing about them is saying: “I don’t know which one is going to be the right one for you, and you may have to try 10 before you get one that suits you.” I wish I could make it easier for people* [laughs]*, but it is a bit of trial and error.’*

(GP 4, male salaried, child with eczema, <1 years’ experience)

*‘I think my knowledge on it’s a bit dodgy* [laughs]*, ‘cos I can never remember how many — how many tubs you’re supposed to get through in a week if you’re using properly.’*

(GP 7, male partner, 2 years’ experience)

Although several GPs found the formularies within clinical systems helped them with prescribing, others complained that systems which automatically recommended switches to more cost-effective products created confusion for parents and clinicians, who were unfamiliar with the alternative products. Sometimes when changes were made, it brought GPs into conflict with parents wanting previously prescribed treatments:

‘Some patients have a particular cream that they want to be prescribed for them that’s not on the formulary or is more expensive, so that can provide a bit of conflict around whether you should or shouldn’t do that as to what the evidence is versus the cost versus patient choice.’

(GP 9, female partner, 11 years’ experience)

All participants were confident prescribing mild and moderate potency TCS in children with eczema. However, there was widespread reluctance to prescribe a potent TCS in children when indicated, unless accompanied by a specialist dermatology referral:

*‘On the body, um, I’d go up to Eumovate^™^* [clobetasone butyrate], *and if I was referring to dermatology I’d go up to Elocon^®^* [mometasone furoate], *but only in the context of doing a derm referral as well.’*

(GP 12, male partner, 4 years’ experience)

The fear of potent steroid use among GPs was evident in the experience of the GPSI below who took referrals from colleagues:

*‘It’s always the stigma of steroid prescriptions which is not helped by my colleagues, which* [sic] *still have that unfortunate thing in their head that the steroids are very dangerous and only prescribe hydrocortisone even if the eczema is quite bad.’*

(GP 2, male partner, GPSI, 16 years’ experience)

Advice given to parents about TCS risks, and using them sparingly, as well as a stepwise approach to potencies, was mentioned by most of the interviewees, although several participants expressed uncertainty about quantities of TCS to prescribe. GPs’ underconfidence with potent TCS is compounded for some by their perception of parental fear of TCS. Some reported a need to address parental misconceptions around TCS safety and the challenging conversations they experienced with parents around TCS use:

‘They worry about side-effects so they go “oh, does that mean that they’re going to get — the skin’s going to be thin, and they’re going to be prone to infection, and is that safe to use on a long-term basis?” and that sort of conversation.’

(GP 8, female salaried, 14 years’ experience)

A minority of participants described managing some patients who they felt were over reliant on TCS:

‘So it’s trying to get them to understand that if they put in the hard graft and use the moisturiser more frequently, they can reduce their steroid use and reserve the steroid for when it’s really necessary.’

(GP 14, female partner, 11 years’ experience)

### Supportive information offered and review

GPs tended to only give limited supportive information, with a minority reporting giving advice about avoidance of triggers and irritants, or instructions on the quantity or application of topical treatments. When written information was offered, the resources most commonly referred to were websites, with only a few GPs providing (informal) written instructions or viewing the prescription as a form of written advice. Several GPs expressed a desire to have greater access to community dermatology nurses, who they felt had more time and expertise to explain treatments:

‘It would be nice if we could refer directly to an eczema nurse or a nurse specialist. I think their wealth of knowledge in terms of practical use is awesome, and I think they’d be a lot better at the management plan and going through stuff with parents.’

(GP 12, male partner, 4 years’ experience)

Review of patients with eczema was a reactive process, in which around half of GPs said they routinely gave safety net advice; that is, to return if their eczema became infected or treatment escalation was needed. Proactive assessment of symptoms and adherence to treatments was uncommon, although many GPs saw their potential value. They attributed the current lack of routine review to pressure on appointments, no financial incentives to undertake such reviews, and shortage of trained nurses to support them:

‘Well, in an ideal world you’d follow them up, but in the busy practice and demands on a patient appointment then I would follow-up if they felt they needed it. No formal follow-up, unless it was absolutely awful and you felt you had to see it.’

(GP 9, female partner, 11 years’ experience)

### GPs’ perception of parental management of eczema

GPs perceived differences to parents regarding the assessment of the severity of eczema, underlying aetiology, and expectations around treatments and/or referrals. GPs thought that parents struggled with their understanding of eczema as a long-term condition:

‘I don’t think they find it difficult to accept that this is called eczema. I think it’s difficult accepting that it will be chronic and they will have a tendency to it, and accepting that it’s not caused by something that they can treat and then it will go away.’

(GP 6, female salaried, 23 years’ experience)

GPs felt online resources and word-of-mouth were the most common sources of advice for parents of children with eczema, with the internet being blamed for misinformation around allergies:

‘There’s a lot of internet misinformation about allergy testing as well, I think.’

(GP 14, female partner, 11 years’ experience)

Most GPs believed that the evidence for food allergy causing eczema was weak, except when the clinical history strongly supported this, or in more severe cases of eczema.

Uncertainty was expressed about how to discuss allergy testing and dietary manipulation with parents:

‘I also find it quite hard to understand myself so it is — I find it quite hard to explain to people in the context of eczema who might and who is unlikely to benefit from allergy testing, and realistically what they can hope to achieve.’

(GP 14, female partner, 11 years’ experience)

In addition, GPs were concerned about the limitations of currently available allergy tests, as well as a shortage of allergy clinic appointments and a desire to conserve resources:

‘Just because they react to it when you stick a needle under their skin with this particular substance doesn’t mean it’s necessarily what’s going to be triggering their eczema when they eat it, or any of their other allergies.’

(GP 1, female partner, 13 years’ experience)

GPs tended to dissuade referral for allergy testing or trials of exclusion diets. Sometimes this was done by explaining their concerns about the limitations of testing, or by simply:

‘... *telling them not to go down that route if they can.*’

(GP 3, female salaried, 20 years’ experience)

A few GPs reported feeling pressured by parents to refer children for allergy testing:

‘Well, it’s difficult, because it depends on the patient, doesn’t it? ‘Cos some of them just want to see an allergist.’

(GP 13, female salaried, 17 years’ experience)

GPs reported that parents struggled to recall the names of previous treatments, making it a challenge for them to keep track of what had been tried. This, together with participants’ perception that parents have difficulty recalling instructions on their use, may be contributing to the confusion with treatments which GPs felt was common in eczema care:

‘Cos some of them are like, ‘oh I don’t know, uh well this tube, the red one, you know’. They don’t remember what the names of their medication — the creams are.’

(GP 3, female salaried, 20 years’ experience)

Records of repeat prescriptions on patients’ electronic medical records were the most commonly reported method used by GPs to assess adherence to treatments, particularly emollients.

Underuse of both emollients and TCS by parents was felt to be common place:

‘When you question people about how they use steroids, they often use dribs and drabs for ever and ever, and not enough emollients.’

(GP 5, female partner, 12 years’ experience)

## DISCUSSION

### Summary

As far as the authors are aware, this is the first study to explore GPs’ experiences of diagnosing and managing children with eczema. Participants described important gaps in training, knowledge, and practice. This was coupled with a feeling that other chronic diseases and older patients took priority. Apart from children with darker skin types or with possible skin infections, diagnosis of eczema was straightforward for most GPs, yet there was uncertainty about prescription quantities, use of potent TCS, and a trial-and-error approach to emollients. Parents were reported to frequently forget medication names and instructions on their use, and low adherence to medications was felt to be commonplace, with reluctance to use TCS. However, support offered to parents by means of verbal and written advice about treatments was limited, and few participants said they routinely arranged reviews to assess treatment acceptability, adherence, and disease control. A particular challenge for GPs was reconciling parents’ concerns about allergy and requests for allergy testing with their understanding that it had no role for the majority of children.

### Strengths and limitations

This sample was diverse with respect to age, years working as a GP, and practice population served. The discussion of real-life cases ensured that the data were grounded in everyday clinical practice and the authors believe that they achieved saturation as no new themes were emerging in the later interviews. However, the predominance of females and GP partners, as well as the self-selection of doctors with professional (specialist interest) or personal interest in eczema, may mean the views of GPs less confident with managing eczema are under-represented. There may also be differences between the ‘public’ accounts given in the interviews and participants’ actual clinical practice. GPs’ views of parents are just that, and may be inaccurate or unconsciously biased. However, this work still provides important insights into professional perceptions and attitudes to parents or carers of children with eczema, and much of it is supported by the existing literature.

### Comparison with existing literature

These results are consistent with previous work which has identified limited training for GPs in dermatology at both undergraduate and postgraduate levels.^21^^,^^22^

The perception among GPs in this study of a low emphasis on dermatology within primary care due to increasing demands of an ageing population with complex comorbidities concurs with a recent qualitative study where GPs reported a larger palliative and chronic disease workload.^23^

In terms of treating and managing eczema in primary care, these findings of a trial and error approach to emollients by GPs are similar to previous studies with parents,^9^ and there may be hidden costs in terms of parent confusion, repeated consultations, and GP–parent conflict when clinical prescribing systems encourage switching to more ‘cost-effective’ products. Although TCS have been shown to have a low risk of causing adverse effects when used appropriately,^24^ TCS ‘phobia’ among parents of children has been widely reported,^10^^,^^25^^,^^26^ mirroring GPs’ views in this study that parents are fearful of using them. However, the authors also found that GPs themselves lack confidence in using potent TCS (despite current British guidelines recommending their use short term on the trunk or limbs in children >12 months when their eczema has not improved with mild-to-moderate potency TCS and secondary infection has been excluded).^27^ The typical advice given by GPs to parents in this study to use TCS sparingly concurs with a previous survey of GPs,^28^ and may be inadvertently contributing to parental fear of their use.

The authors have recently published research that found a mismatch between GP and parent assessment of eczema severity,^6^ and the 2007 NICE guidelines^27^ flagged up that healthcare professionals can underestimate the psychosocial impact of the condition. Participants’ perception that parents are seeking an underlying cause for their child’s eczema, and struggle to source reliable online information, concurs with previous surveys and qualitative studies with parents.^9^^,^^29^^,^^30^ GPs in this study expressed uncertainty about referral for allergy testing or trial of exclusion diets which reflects both the controversy around the role of allergy in eczema,^31^^,^^32^ the limitations of allergy testing currently available,^33^^,^^34^ and the poor evidence base for exclusion diets in patients with eczema.^34^^,^^35^ Despite the lack of evidence, dietary manipulation by parents is common, with up to 75% of parents trying dietary exclusions (49% unsupervised), and 41% dietary supplementation.^36^ Similar findings to those in this study, of a lack of parental understanding of disease pathogenesis and treatment regimens, have also been previously identified.^37^ The differing perspectives and beliefs held by clinicians and parents undermines a solid basis for establishing a shared understanding and treatment plan.

### Implications for research and practice

Although the participants in this study probably had an above-average interest and knowledge about eczema, this study highlights that the common, impactful primary care condition of eczema is accorded low priority in the context of high competing demands, and GPs’ knowledge and confidence in managing it could be improved. Specifically, GPs need to be aware of and acknowledge the psychosocial impact of eczema,^38^ and both understand and communicate the safe use of TCS. Confident use of TCS by GPs should encourage parents to be more comfortable with their safety, thereby helping reduce ‘steroid phobia’. Issuing prescriptions with both TCS potency information and fingertip unit directions on use are simple changes that could aid patient understanding of dosing.^39^ National guidelines recommend proactive follow-up,^27^ and financial rewards to incentivise GPs to do this should be considered. Reviews may improve adherence,^40^^,^^41^ and could also reduce previously reported parental perception of feeling dismissed by their healthcare provider.^9^

Future research could further explore the extent to which stated barriers to better care of children with eczema are a product of system factors (for example, lack of time, limited access to allergy services) or attitudinal factors (for example, low regard for impact of the condition on the child and their family). While there is uncertainty over the clinical and cost-effectiveness of interventions to improve the self-management of people with eczema,^4^ WAPs and signposting to high quality information are advocated^27^^,^^42^ to improve understanding and adherence with treatments. Within the context of the authors’ wider study looking at developing an eczema action plan, the GPs were generally supportive of such a tool.^15^

Action plans may be one way to help address the information gap between GP and parent. However, further research is needed to evaluate the acceptability and clinical-and cost-effectiveness of WAPs for children with eczema in daily clinical practice. Future research should also consider the role of the practice nurse, which could be extended to include the review and support of families with eczema, as happens with the related atopic long-term condition of asthma. Allergy testing and food avoidance is an area of controversy in eczema and has been identified as a key area requiring further research,^43^ which is needed to clarify when investigation is most likely to benefit affected children and their families.
